# Anti-inflammatory and anti-excitoxic effects of diethyl oxopropanamide, an ethyl pyruvate bioisoster, exert robust neuroprotective effects in the postischemic brain

**DOI:** 10.1038/srep42891

**Published:** 2017-02-21

**Authors:** Hye-Kyung Lee, Il-Doo Kim, Seung-Woo Kim, Hahnbie Lee, Ju-Young Park, Sung-Hwa Yoon, Ja-Kyeong Lee

**Affiliations:** 1 Department of Anatomy, Inha University School of Medicine, Inchon, Republic of Korea; 2 Medical Research Center, Inha University School of Medicine, Inchon, Republic of Korea; 3 Department of Biomedical Sciences, Inha University School of Medicine, Inchon, Republic of Korea; 4 Department of Molecular Science and Technology, Ajou University, Suwon, Republic of Korea

## Abstract

Ethyl pyruvate (EP) is a simple aliphatic ester of pyruvic acid and has been shown to have robust neuroprotective effects via its anti-inflammatory, anti-oxidative, and anti-apoptotic functions. In an effort to develop novel EP derivatives with greater protective potencies than EP, we generated four EP isosteres, among them the neuroprotective potency of N,N-diethyl-2-oxopropanamide (DEOPA), in which the ethoxy group of EP was replaced with diethylamine, was far greater than that of EP. When DEOPA was administered intravenously (5 mg/kg) to rat middle cerebral artery occlusion (MCAO) model at 6 hrs post-surgery, it suppressed infarct formation, ameliorated neurological and sensory/motor deficits, and inhibited microglial activation and neutrophil infiltrations in the postischemic brain more effectively than EP. In particular, DEOPA markedly suppressed LPS-induced nitrite production and cytokine/chemokine inductions in microglia, neutrophils, and endothelial cells and these effects are attributable to inhibition of the activity of NF-κB by suppressing IκB-α degradation and p65 to DNA binding. In addition, DEOPA suppressed NMDA-induced neuronal cell death in primary cortical neuron cultures by NAD replenishment and suppression of NF-κB activity. Together, these results indicate DEOPA has multi-modal protective effects against ischemic brain damage targeting numerous cell types in the brain and also against other inflammation-related diseases.

Ethyl pyruvate (EP) is a simple aliphatic ester of pyruvic acid and has been reported to mitigate the damage caused by various stressors, such as, hemorrhagic shock, stroke, sepsis, and acute pancreatitis^1^,^2^,^3^,^4^. Accumulating evidences indicates EP is a multi-functional protective agent that possesses anti-inflammatory, anti-oxidative, anti-apoptotic, and ion-chelating effects^5^,^6^,^7^,^8^. Its anti-inflammatory effects have been studied by many researchers, and it has been reported to play important roles in the above-mentioned pathological conditions. Various molecular mechanisms have been proposed to underlie the anti-inflammatory effects of EP. These include the suppression of NF-κB activity^9^,^10^, the suppression of the secretion/release of High mobility group box-1 (HMGB1, a danger-associated molecular pattern molecule)^8^,^11^,^12^. Regarding its anti-oxidative effects, EP reduces ROS production and promotes the decomposition of H_2_O_2_ in dopamine-treated PC12 cells^13^. We have previously reported an interrelationship between the anti-inflammatory and anti-oxidative effects of EP in that EP-mediated Nrf2 translocation in BV2 cells (a microglia cell line) and subsequent interaction between Nrf2 and p300 to suppress the p65-p300 interaction^14^. In terms of its ion chelating effects, we previously suggested the neuroprotective effect of EP against Zn^2+^ toxicity might be due to two effects, namely, NAD replenishment and direct Zn^2+^ chelation^15^. In addition, we also found direct Ca^2+^ chelation by EP suppresses the phosphorylation and secretion of HMGB1 in microglia^8^. Therefore, EP appears to have a variety of protective effects that might be conveyed directly by EP or by pyruvate produced by the metabolization of EP.

Protective effects of EP have been documented in various diseases that affect the central nervous system (CNS). For example, EP suppressed microglia activation and inflammatory marker inductions, significantly reduced infarct volumes, and mitigated neurological deficits in rat model of middle cerebral artery occlusion (MCAO)^4^ and attenuated kainic acid-induced neuronal cell death in the CA1 and CA3 regions of the mouse hippocampus^16^. EP also suppressed the death of nigrostriatal dopaminergic (DA) neurons in the mouse model of Parkinson’s disease^17^ and improved motor function scores in models of spinal cord ischemia and traumatic brain injury^18^,^19^. The anti-inflammatory and anti-oxidative effects of EP exhibited robust neuroprotective effects in above-mentioned diseases of the CNS.

In an effort to develop more effective therapeutics based on EP, many researchers have screened or generated derivatives of EP or of pyruvate. Sappington *et al*.^20^ screened 15 commercially available EP-related compounds for cytoprotective and anti-inflammatory effects and reported the anti-inflammatory effects of diethyl oxaloproprionate (DEOP) and 2-acetamidoacrylate (2AA) were comparable to those of EP, and that methyl 2-acetamidoacrylate (Me-2AA; a methyl derivative of 2AA) had greater anti-inflammatory effects than EP or 2AA. However, when two α-keto-carboxylic acid derivatives of EP were examined, their protective potencies were less than those of EP in a rat model of multivisceral ischemia-reperfusion^21^. Because, many beneficial effects of EP are common to pyruvate, for example, NAD replenishment and hydroxyl radical scavenging^15^,^22^, in the present study, we generated four EP isosteres by saving the pyruvoyl group and replacing the ethoxy group of EP with ethylamine, diethylamine, thioester, or n-propyl to form the corresponding amides. We subsequently evaluated the neuroprotective effects of these isosteres in our rat model of MCAO and sought to identify the molecular mechanisms responsible for their effects in this model and in cell lines for microglia, neutrophil, and endothelial cells and primary cortical cultures.

## Results

### Neuroprotective potencies of the four EP isosteres in postischemic brains

**Figure 1:**
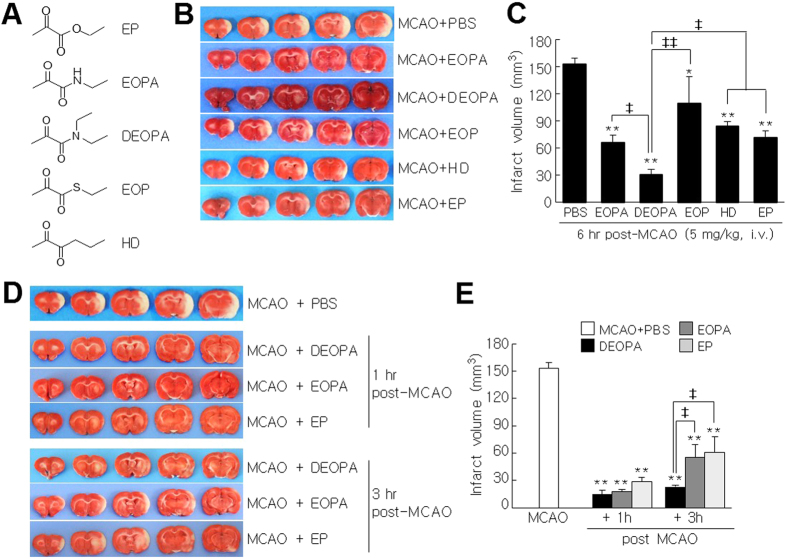
Infarct suppressions by the four EP isosteres. (**A**) Structures of EP and its isosteres, N-ethyl-2-oxopropanamide (EOPA), N,N-diethyl-2-oxopropanamide (DEOPA), S-ethyl-2-oxopropanethioate (EOP), and n-hexane-2,3-dione (HD). (**B**,**C**) The five agents were administered intravenously (all at 5 mg/kg) at 6 hrs post-MCAO. (**D**,**E**) DEOPA, EOPA, or EP (all at 5 mg/kg) were administered intravenously at 1 or 3 hrs post-MCAO. Mean infarct volumes were assessed at 2 days post-MCAO by TTC staining. Representative images of infarctions in coronal brain sections (**B,D**) and quantitative results (means ± SEMs) (**C,E**). MCAO + PBS, PBS-treated MCAO control animals (n = 8); MCAO + EOPA, EOPA-administered MCAO animals (n = 10); MCAO + DEOPA, DEOPA-administered MCAO animals (n = 10); MCAO + EOP, EOP-administered MCAO animals (n = 4); MCAO + HD, HD-administered MCAO animals (n = 4); MCAO + EP, EP-administered MCAO animals (n = 10). *p < 0.05, **p < 0.01 vs. the MCAO group, ^‡^*p* < 0.05, ^‡‡^*p* < 0.01 vs. the MCAO + DEOPA group.

To examine the neuroprotective effects of the four EP isosteres (Fig. 1A), N-ethyl-2-oxopropanamide (EOPA), N,N-diethyl-2-oxopropanamide (DEOPA), S-ethyl-2-oxopropanethioate (EOP), and hexane-2,3-dione (HD), 5 mg/kg of each isoster was administered intravenously at 6 hrs post-MCAO and infarct volumes were assessed 2 days after surgery. Infarct volumes in MCAO + EOPA, MCAO + DEOPA, MCAO + EOP, and MCAO + HD groups were reduced to 43.1 ± 5.5% (n = 5, p < 0.01), 20.3 ± 3.8% (n = 5, p < 0.01), 71.6 ± 19.0% (n = 5, p < 0.01), and 55.1 ± 3.2% (n = 4, p < 0.01), respectively, of that of PBS-treated MCAO controls (Fig. 1B and C). Since the administration of 5 mg/kg of EP i.v. at 6 hrs post-MCAO reduced infarct volume to 46.8 ± 5.2% (n = 5, p < 0.01) (Fig. 1B and C), DEOPA was found to have a significantly greater neuroprotective effect than EP, whereas the neuroprotective effect of EOPA was similar to that of EP. Similar results were obtained when the neuroprotective potencies of DEOPA, EOPA, and EP were compared after administering them at 1 or 3 hrs post-MCAO, i.v., DEOPA was found to have a significantly greater neuroprotective effect than EP, whereas the neuroprotective effect of EOPA was similar to that of EP.

### Neuroprotective potency of DEOPA in the postischemic brain

**Figure 2:**
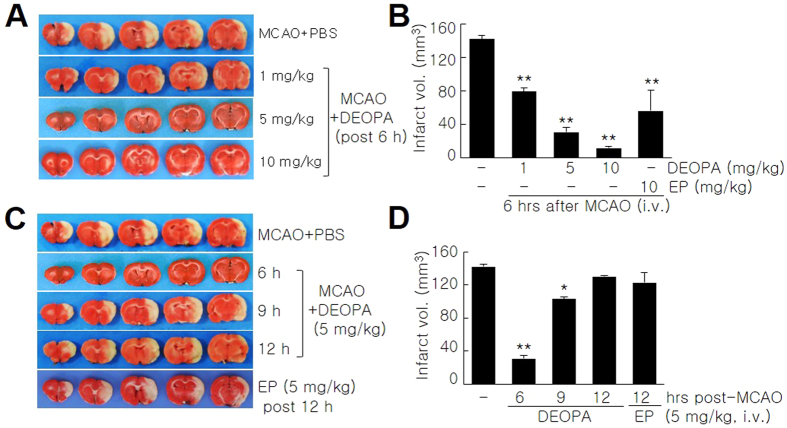
Neuroprotective effects of DEOPA in the postischemic brain. (**A,B**) DEOPA (1, 5, or 10 mg/kg) was administered intravenously at 6 hrs post-MCAO (n = 5) and mean infarct volumes were assessed at 2 days post-MCAO by TTC staining. Representative images of infarctions in coronal brain sections (**A**) and mean infarct volumes at 2 days post-MCAO (means ± SEMs; n = 5) (**B**). (**C**,**D**) DEOPA (5 mg/kg) was administered intravenously at 6, 9, or 12 hrs post-MCAO (n = 5–6) and mean infarct volumes were assessed at 2 days post-MCAO by TTC staining (**C**). Results are presented as means ± SEMs (n = 5–6) (**D)**. MCAO + PBS, PBS-treated MCAO control animals; MCAO + DEOPA, the DEOPA-administered MCAO animals; MCAO + EP, the EP-administered MCAO animals. *p < 0.05, **p < 0.01 vs. the MCAO group.

To examine the neuroprotective potency of DEOPA in more detail, 1, 5, or 10 mg/kg of DEOPA was administered i.v. at 6 hrs post-MCAO and infarct volumes were assessed at 2 days after surgery. Mean infarct volumes were reduced to 55.7 ± 3.0% (n = 5, p < 0.01), 20.0 ± 3.8% (n = 5, p < 0.01), and 8.4 ± 1.0% (n = 5, p < 0.01), respectively, versus PBS-treated MCAO controls (Fig. 2A and B). Importantly, the reduction of infarct volume achieved by 10 mg/kg of DEOPA administered at 6 hrs post-MCAO was greater than that achieved by the same amount of EP (Fig. 2A and B). In addition, 5 mg/kg of DEOPA administered at 9 or 12 hrs post-MCAO reduced mean infarct volumes to 72.4 ± 2.8% (n = 6, p < 0.05) and 91.9 ± 1.5% (n = 5), respectively (Fig. 2C and D). These results confirmed DEOPA had greater neuroprotective potency than EP in the postischemic rat brain.

### Suppressions of neurological deficits and motor impairments by DEOPA

**Figure 3:**
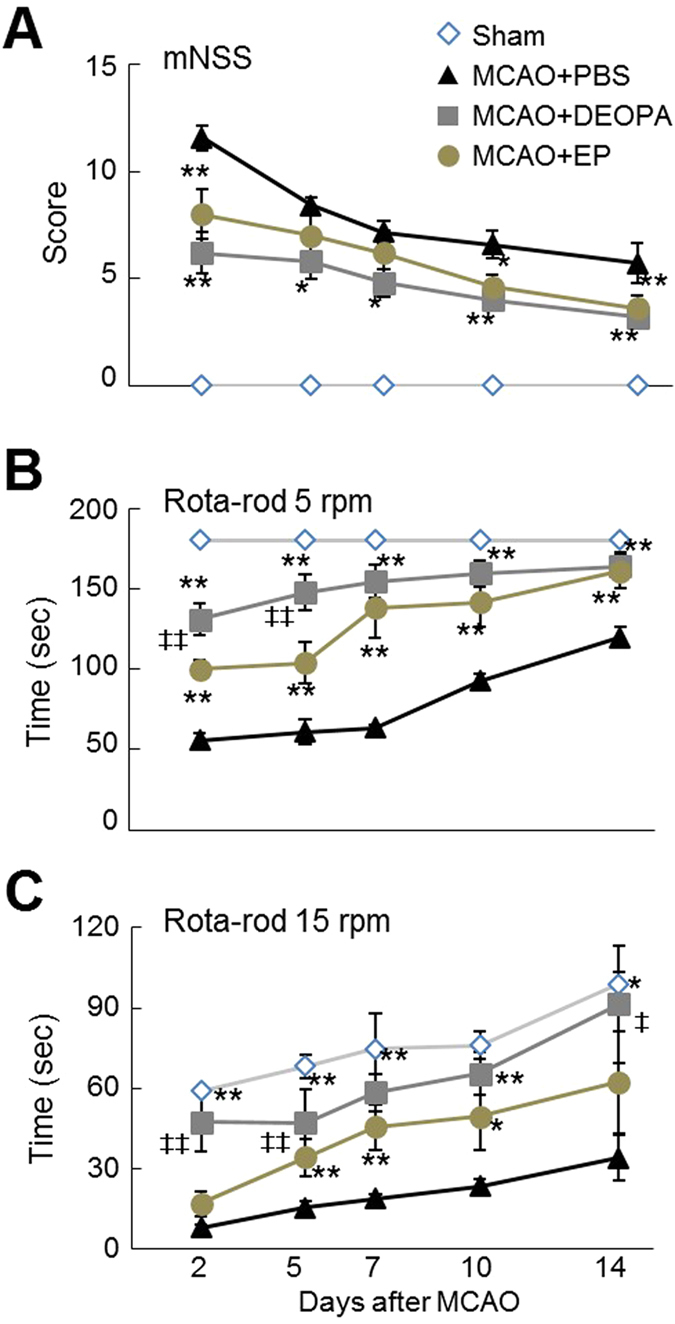
Amelioration of motor deficit by DEOPA. DEOPA (5 mg/kg) was administered 6 hrs post-MCAO and neurological deficits were evaluated using modified neurological severity scores at 2, 5, 7, 10, or 14 days post-MCAO (**A**). The rota-rod test was performed at 5 or 15 rpm at 2, 5, 7, 10, or 14 days post-MCAO with a 1 hr rest period between tests (**B**,**C**). Sham, sham-operated animals; MCAO + PBS, PBS-treated MCAO control animals; MCAO + DEOPA, DEOPA-administered MCAO animals; MCAO + EP, EP-administered MCAO animals. Results are presented as mean ± SEM (n = 8). *p < 0.05, **p < 0.01 versus treatment naïve-MCAO controls, ^‡^*p* < 0.05, ^‡‡^*p* < 0.01 vs. DEOPA-administered MCAO group.

To determine whether DEOPA could improve neurological deficits and motor impairment, 5 mg/kg of DEOPA was administered 6 hrs post-MCAO and modified neurological severity scores (mNSSs) were measured from 2 to 14 days post-MCAO. In the MCAO + DEOPA group, mean modified neurological severity score (mNSS) was 6.2 ± 0.9 (n = 8, p < 0.01) at 2 days post-MCAO, which was significantly lower than that of PBS-treated MCAO controls (11.5 ± 0.5, n = 8) (Fig. 3A). Furthermore, these improvements in the MCAO + DEOPA group were maintained until 14 days post-MCAO (Fig. 3A). When motor activities were assessed by rota-rod testing at 5 rpm, mean latency (time spent on the rod) in the MCAO + DEOPA group was significantly greater than that of PBS-treated MCAO controls (MCAO + PBS) until 14 days (Fig. 3B). Mean latency in the MCAO + DEOPA group was significantly greater than that of MCAO + EP group until 5 days, although this difference disappeared at 7 days (Fig. 3B). Interestingly, at 15 rpm, mean latencies in the MCAO + DEOPA was significantly greater than that of PBS-treated MCAO controls throughout the experimental period until 14 days or of MCAO + EP group, in particular at 2, 5, and 14 days post-MCAO (Fig. 3B). These results suggest that the marked infarct suppression observed for DEOPA was accompanied by better neurological and motor outcomes, and that these effects were evident by a task with a high degree of difficulty and at delayed time points. Furthermore, pH, PaO_2_, PaCO_2_, and blood glucose levels were similar in the MCAO + DEOPA and the PBS-treated MCAO group, indicating that physiological parameters were not influenced by DEOPA (Supplementary Table S1).

### Anti-inflammatory effects of DEOPA in the postischemic brain

**Figure 4:**
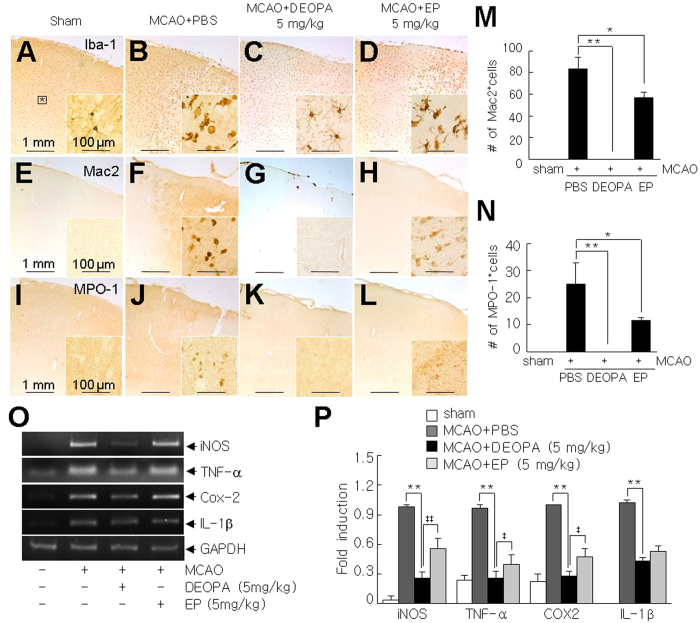
Suppression of inflammatory processes by DEOPA in the postischemic brain. (**A**–**L**) Coronal brain sections were obtained 2 days after surgery in the sham (**A,E,I**), MCAO + PBS (**B,F,J**), MCAO + DEOPA (**C,G,K**), and MCAO + EP (**D,H,L**) groups. Activated microglia were stained using anti-Iba-1 (**A–D**), anti-Mac-2 (**E–H**), or anti-MPO-1 (**I–L**) antibodies. The insets (**A–L**) are high magnification photographs of the indicated regions (*) in A. Photographs are representative of three independent experiments. Scale bars in A-L represent 1 mm and those in insets represent 100 μM. (**M**,**N**) Numbers of Mac-2^+^ and MPO-1^+^ cells in indicated regions in A (*) (0.1 mm^2^) were counted. Counts are presented as means ± SEMs (n = 12 from 3 animals). (**O-P**) RT-PCR samples were prepared from the indicated region (the black box in **A**). The RNA levels of pro-inflammatory markers in the experimental groups were assessed at 2 days post-MCAO. Changes in the RNA levels of iNOS, Cox-2, TNF-α, and IL-1β are presented as means ± SEMs (n = 3). Sham, sham-operated rats; MCAO + PBS, PBS-treated MCAO control rats; MCAO + DEOPA, DEOPA-administered MCAO rats; MCAO + EP, EP-administered MCAO rats. *p < 0.05, **p < 0.01 vs. the MCAO group, ^‡^*p* < 0.05, ^‡‡^*p* < 0.01 between indicated groups.

Since EP is known to have strong anti-inflammatory effects, we examined anti-inflammatory potency of DEOPA in the postischemic brain. DEOPA (5 mg/kg, i.v.) was administered at 6 hrs post-MCAO and brain sections obtained at 2 days post-MCAO were stained with antibody against Iba-1 (a marker of cells of myeloid origin), Mac2 (a marker of activated resident microglia), or MPO-1 (myeloperoxidase-1, neutrophil marker). In sham controls, Iba-1^+^ cells exhibited a ramified morphology and were detected throughout brains (Fig. 4A). However, at 2 days post-MCAO, Iba-1^+^ cells in the cortices of ipsilateral hemispheres of PBS-treated MCAO controls were amoeboid in shape, indicating an activated state (Fig. 4B). However, in the MCAO + DEOPA group, most Iba-1^+^ cells retained a ramified morphology (Fig. 4C). In contrast to Iba-1^+^ cells, Mac2^+^ and MPO-1^+^ cells were barely detected in sham controls (Fig. 4E and I), whereas in treatment naïve-MCAO controls, numbers of these cells were markedly elevated at 2 days post-MCAO (Fig. 4F, J, M and N). However, they were hardly detected in the MCAO + DEOPA group (Fig. 4G, K, M and N). Notably, the suppressive effects of DEOPA were greater than those of EP in both staining (Fig. 4H and L–N). In addition, the inductions of proinflammatory markers (iNOS, TNF-α, Cox-2, IL-1β) observed at 2 days post-MCAO in the PBS-treated MCAO group were significantly suppressed in the MCAO + DEOPA group to an extent greater than that was observed in the MCAO + EP group (Fig. 4O and P). These results indicated the anti-inflammatory potency of DEOPA was greater than EP.

### Suppressions of NO and of proinflammatory marker inductions in activated microglia by DEOPA

**Figure 5:**
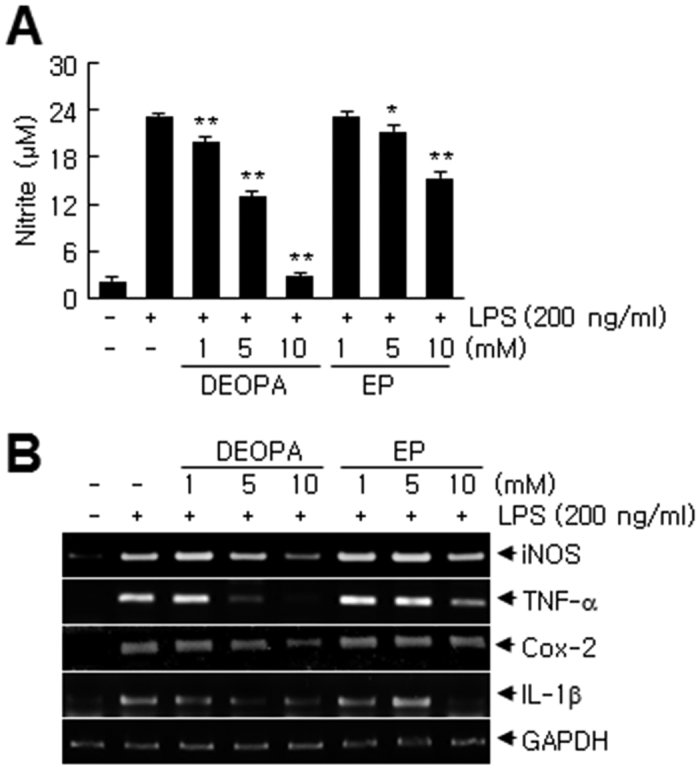
Suppression of the LPS-induced activation of BV2 cells by DEOPA. Nitrite production (**A**) and expressions of proinflammatory markers (**B**) were assessed using a Griess assay and by RT-PCR, respectively. BV2 cells (1.5 × 10^5^ cells/well in 24-well culture dishes) were co-treated with DEOPA or EP (1, 5, or 10 mM) for 1 hr, washed, and incubated with LPS (200 ng/ml) for 24 hrs. Changes in nitrite levels are presented as means ± SEMs. *p < 0.05, **p < 0.01 vs. LPS treatment alone.

To confirm the anti-inflammatory effects of DEOPA, BV2 cells (a microglia cell line) were treated with LPS (200 ng/ml) for 24 hrs and nitrite levels were measured in the presence or absence of DEOPA. NO production was dose-dependently suppressed by co-treating DEOPA (1, 5, or 10 mM) and reduced to the basal level at 10 mM (Fig. 5A). Importantly, the efficacy of DEOPA was greater than that of EP at all doses tested (Fig. 5A). Similarly, iNOS induction was markedly suppressed by DEOPA (5 or 10 mM) and these suppressive effects were also greater than those achieved by the same doses of EP (Fig. 5B). Furthermore, proinflammatory marker (TNF-α, Cox-2, and IL-1β) levels induced by LPS were also markedly suppressed by DEOPA, and once again the suppressive effects of DEOPA were greater than those of EP at all doses tested (Fig. 5B).

### Suppression of NF-κB activity by DEOPA via the inhibition of IκB-α degradation in cytosol and of p65 to DNA binding in nucleus

**Figure 6:**
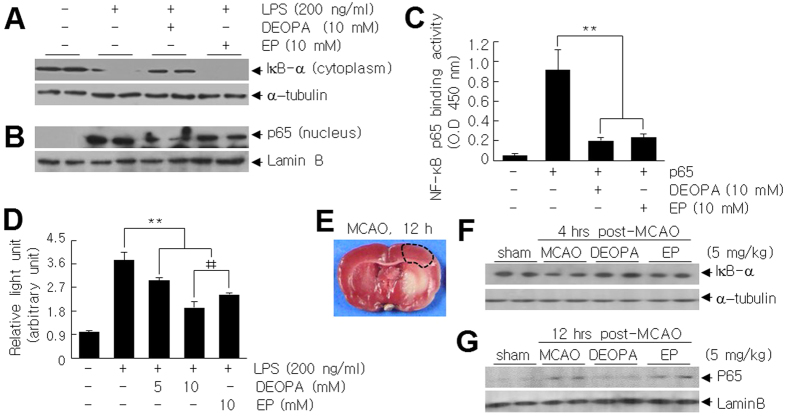
Suppression of NF-kB activity by DEOPA in LPS-treated BV2 cells. (**A,B**) Effects of DEOPA (10 mM) or EP (10 mM) on cytoplasmic IκB levels (**A**) and on nuclear p65 levels (**B**) in BV2 cells were examined by immunoblotting after treating cells with LPS (200 ng/ml) for 15 min or 1 hr, respectively. Alpha-tubulin and lamin B were used as loading controls. (**C**) NF-κB binding was assessed using TransAM p65 assay kit and recombinant p65 in the presence or absence of DEOPA (10 mM) or EP (10 mM). (**D**) NF-κB activities in the presence or absence of DEOPA (5 or 10 mM) or of EP (10 mM) were examined in BV2 cells transfected with NF-κB-Luc reporter plasmid after treatment with LPS for 1 hr. NF-κB activities are presented as means ± SEMs (n = 4), **p < 0.01, ^‡‡^p < 0.01 between indicated group. (**E**–**G**) Effects of DEOPA (5 mg/kg) or EP (5 mg/kg) on cytoplasmic IκB levels (**F**) and on nuclear p65 levels (**G**) in cortical penumbra of the ischemic hemisphere (**E**) were examined at 4 or 12 hrs post-MCAO, respectively, by immunoblotting after administering them at 1 hr post-MCAO.

The marked suppression of nitrite production and proinflammatory marker induction by DEOPA (Fig. 5) prompted us to examine the effects of DEOPA on the NF-κB signaling pathway. The amount of IκB-α in the cytoplasm of LPS-treated BV2 cells was significantly decreased after treatment with LPS (200 ng/ml) for 15 min and this decrease was suppressed by pre-treating DEOPA (10 mM) but not by pre-treating EP (10 mM) (Fig. 6A). Consistent with these observations, the nuclear translocation of p65 was markedly suppressed by DEOPA but not by EP (Fig. 6B), indicating DEOPA suppressed the nuclear translocation of p65 probably by inhibiting IκB-α degradation in cytosol. Under these conditions, levels of α-tubulin and lamin B were unchanged in cytoplasm and nucleus, respectively (Fig. 6A and B). In addition, when the DNA binding activity of p65 was examined using the TransAM NF-κb p65 assay kit and recombinant p65, LPS-induced p65 to DNA binding activity was found to be suppressed by DEOPA and by EP, and the suppressive effect of DEOPA was comparable to EP (Fig. 6C). Furthermore, when we measured LPS-induced NF-κB activity after transfecting BV2 cells with NF-kB-Luc reporter plasmid containing five copies of the NF-κB consensus sequence (GGGAATTTCC), NF-κB activity was suppressed by pre-treating DEOPA (10 mM) to 45.2 ± 4.0% (n = 4) of that observed for LPS-induced cells, and this suppression was significantly greater than that obtained by pre-treating the same amount of EP (68.1 ± 4.7%, n = 4; Fig. 6D). Importantly, in the postischemic brain, the decrease of the cytoplasmic IκB-α amounts and the induction of nuclear translocation of p65 were observed in the cortical penumbra of the ischemic hemisphere at 4 or 12 hrs post-MCAO, respectively (Fig. 6E–G). They were significantly suppressed by DEOPA (5 mg/kg) administered at 1 hr post-MCAO (Fig. 6E–G), further confirming these modulations also occurred *in vivo*. These results indicate that DEOPA exerted a robust anti-inflammatory effect by suppressing NF-κB activity at two points, by inhibition of IκB-α degradation in cytoplasm and p65 to DNA binding in nuclei.

### Blockades of neutrophil-endothelial adhesion and of transendothelial neutrophil migration by DEOPA

**Figure 7:**
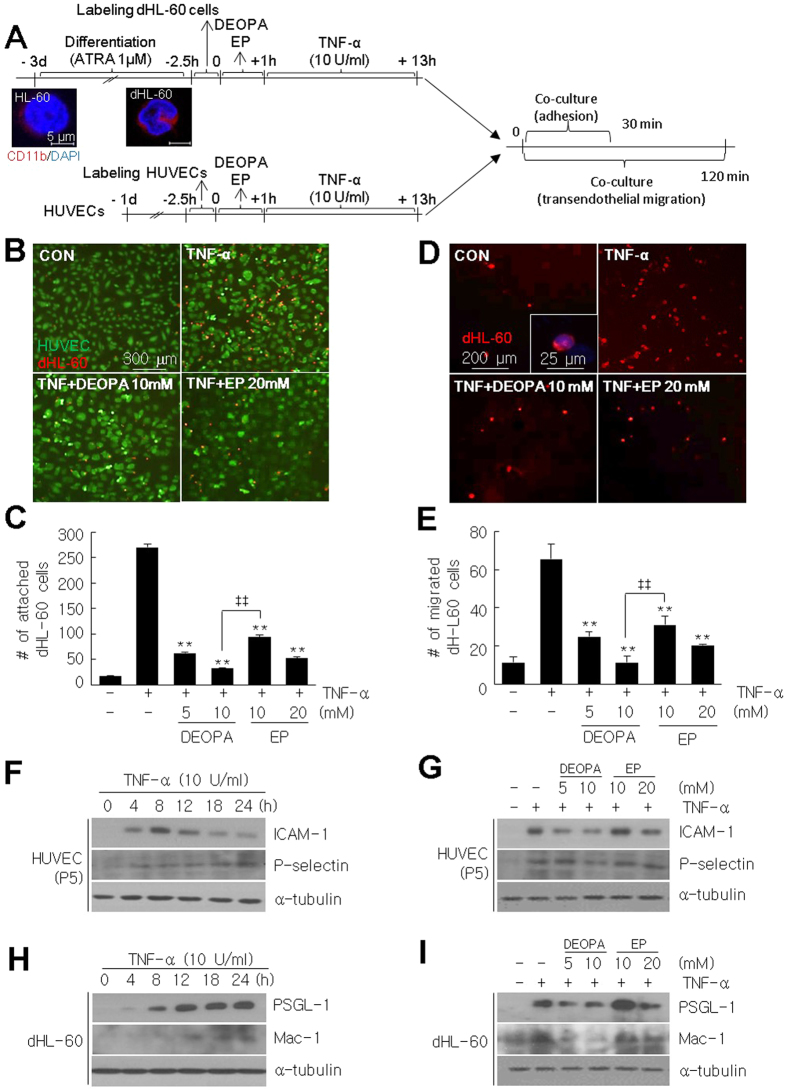
Suppressions of the neutrophil to endothelial adhesion and transendothelial migration and of chemokine and cell adhesion molecule inductions by DEOPA. (**A**) HUVECs were plated on gelatin-coated cover slides and differentiation of HL-60 cells were induced by treating cells with 1 μM of ATRA for 3 days. HUVEC and dHL-60 cells were lableded with CellTracker™ Green CMFDA or Red CMTPX Dye, respectively, before assays. dHL-60 cells were also treated with TNF-α (10 U/ml) for 12 hrs and then added to HUVECs monolayers. DEOPA (5 or 10 mM) or EP (10 or 20 mM) was pre-treated for 1 hr before TNF-α treatment. (**B**–**E**) After 30 min or 2 hrs of co-culture, cells were washed with chilled PBS three times, fixed, and counted the numbers of attached (**B,C**) or transendothelially migrated (**D,E**) dHL-60 cells, respectively. Results are presented as means ± SEMs (n = 3). Scale bars in B and D represent 300 or 200 μm and the one in inset represents 25 μm. **p < 0.01 vs. TNF-α-treated control cells; ^‡‡^p < 0.01 between indicated groups. (**F,H**) Levels of P-selectin and ICAM-1 in HUVECs and of PSGL-1 and Mac-1 in dHL-60 cells were determined by immunoblotting at indicated times after TNF-α (10 U/ml) treatment. (**G,I**) Levels of the same adhesion molecules in HUVECs and in dHL-60 cells were determined by immunoblotting after 12 hrs of TNF-α (10 U/ml) treatment with or without pre-treating DEOPA (5 or 10 mM) or EP (10 or 20 mM).

The almost complete absence of neutrophils in cortical penumbra of MCAO + DEOPA animals (Fig. 4K and N) prompted us to investigate whether DEOPA-mediated suppression of NF-κB activity is also responsible for blocking neutrophil infiltration. Differentiated human promyelocytic leukemia cells (dHL-60 cells) and human umbilical vein endothelial cells (HUVECs) were activated by treating them with TNF-α (10 U/ml) for 12 hrs (Fig. 7A). dHL-60 cells displayed multi-lobular nuclear morphology, indicative of the differentiated state, which was confirmed by staining with anti-CD11 antibody (Fig. 7A). After 30 min of co-culture of dHL-60 cells and HUVECs, the number of dHL60 cells that attached to HUVECs was increased almost 15 fold as compared with TNF-α-untreated cells (Fig. 7B and C). However, pre-treatment of dHL-60 cells and HUVECs with DEOPA (5 or 10 mM) before TNF-α treatment significantly reduced attached cell numbers (Fig. 7B and C). Pre-treatment of EP (10 or 20 mM) also reduced attached cell numbers but to a lesser extent (Fig. 7B and C). Similarly, the numbers of dHL-60 cells that migrated through HUVECs were markedly reduced by pre-treating both cells with DEOPA (10 mM) and these suppressive effects were greater than that of the same amount of EP (Fig. 7D and E). These results suggest DEOPA inhibited neutrophils/endothelial interactions and the transendothelial cell migration of neutrophils.

### Suppression of cell adhesion molecule inductions in endothelial cells and neutrophils by DEOPA

ICAM-1 and P-selection are known to be induced in endothelial cells during neutrophil-endothelial interactions^23^ and modulated by NF-kB^24^,^25^. Expressions of ICAM-1 and P-selection were significantly increased in TNF-α-treated HUVECs and these inductions were effectively suppressed by pre-treating DEOPA (5, 10 mM) for 1 hr before TNF-α treatment (Fig. 7F and G). Similarly, inductions of PSGL-1 and Mac-1 in dHL-60 cells, that binds to endothelial P-selectin^26^ and ICAM-1^27^, respectively, were significantly suppressed by pre-treating DEOPA (5, 10 mM) (Fig. 7H and I). Interestingly, EP also suppressed the upregulations of those genes but the suppressive potencies of DEOPA were greater than EP in all cases (Fig. 7G and I). Together these results indicate that the DEOPA-mediated suppression of NF-κB activity and subsequent inhibitions of cell adhesion molecule expressions might be responsible for blocking neutrophil infiltration into the postischemic brain.

### Anti-excitotoxic effects of DEOPA in primary cortical cultures

**Figure 8:**
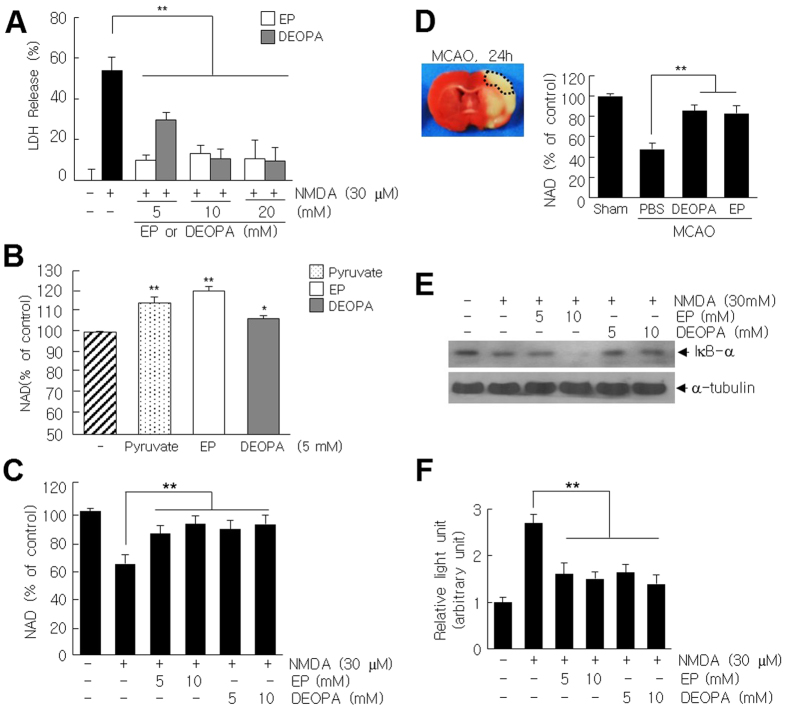
Suppressions of NMDA-induced neuronal cell death by DEOPA. (**A**) LDH levels in primary cortical cultures (4 × 10^5^ cells/well in 24-well) were measured 24 hrs after treatment with NMDA (30 μM, 10 min). (**B**) Levels of NAD in normal primary cortical cultures were measured after 3 hrs of DEOPA, EP, and pyruvate (5 mM of each) treatment. (**C**) Relative NAD levels were measured 24 hrs after treating cells with NMDA (30 μM) for 10 min in the presence or absence of DEOPA or EP (5 or 10 mM of each). (**D**) Relative NAD levels in cortical penumbra of the ischemic hemisphere were measured 24 hrs post-MCAO after administrating DEOPA (5 mg/kg) or EP (5 mg/kg) at 1 hr post-MCAO. (**E**) Cytoplasmic IκB levels were examined by immunoblotting at 24 hrs after co-treating NMDA (30 μM) and EP or DEOPA (5 or 10 mM of each) for 1 hr. (**F**) NMDA (30 μM, 1 hr)-induced NF-κB activities in the presence or absence of DEOPA (5 or 10 mM) or of EP (10 mM) were examined in primary cortical cultures after transfecting them with NF-κB-Luc reporter plasmid. Results are presented as means ± SEMs. *p < 0.05, **p < 0.01 between indicated groups.

We next investigated whether DEOPA protects neurons from acute damaging processes after ischemic/reperfusion, such as, excitotoxicity and Zn^2+^-toxicity. In primary cortical cultures, NMDA (30 μM, 10 min)-induced LDH release was suppressed by EP and DEOPA (5, 10, or 20 mM) (Fig. 8A). Although, at 5 mM, the anti-excitotoxic effect of EP was greater than that of DEOPA, at 10 or 20 mM, anti-excitotoxic efficacies of DEOPA were comparable to those of EP (Fig. 8A). Interestingly, DEOPA did not protect neurons from Zn^2+^-induced toxicity in acute (300 μM, 30 min)- or chronic (40 μM, 24 hrs) Zn^2+^-treated condition, whereas EP did (Supplementary Figure S1)^15^. Since NMDA receptors regulate neuronal PARP-1 expression and activity^28^ and PARP-1 causes cell death by depleting NAD and ATP^29^, we examined NAD levels in NMDA-treated primary cortical cultures in the presence or absence of DEOPA. DEOPA (5 mM) enhanced basal NAD levels in normal primary cortical cultures, although the effect was weaker than those of EP (5 mM) or pyruvate (5 mM) (Fig. 8B). In addition, NMDA (30 μM, 10 min)-induced NAD depletion was significantly suppressed by co-treating DEOPA (5 mM) with NMDA for 10 min, and DEOPA was as effective as EP (5 mM) (Fig. 8C). Interestingly, NAD level was decreased in ischemic hemispheres to 49.5% of that of contralateral hemisphere at 24 hrs after MCAO and it was also restored by DEOPA (5 mg/kg i.v.) to 88.9% of the control (Fig. 8D), further confirmed that the suppression of NAD depletion by DEOPA contributes to a robust neuroprotective effect of DEOPA in the postischemic brain. Moreover, DEOPA also inhibited IκB-α degradation in cytoplasm and suppressed NMDA-induced NF-κB activation in primary cortical cultures (Fig. 8E and F). As it was found in LPS-treated BV2 cells, EP failed to inhibit IκB-α degradation in cytoplasm (Fig. 8E). These results indicate DEOPA has an anti-excitotoxic effect, and suggest this also contributes to its protective effects in the postischemic rat brain.

## Discussion

EP has been shown to have anti-inflammatory, anti-oxidative, and anti-apoptotic effects, and to chelate several metal ions. During our continues efforts to develop novel therapeutics with improved efficacies based on EP, we synthesized four isosteres of EP and found that one of these isosteres, DEOPA, in which the ethoxy group of EP was replaced with diethylamine, was more neuroprotective than EP, more specifically, it suppressed infarct formation and mitigated neurological deficits more effectively than EP in the postischemic brain. Intriguingly, the anti-inflammatory activity of DEOPA was superior to EP and it was attributed to its inhibition of NF-κB activity, which was considerably greater than EP. Interestingly, marked suppression of NF-κB activity was responsible for suppressing activation of microglia and for anti-excitotoxic effect in neurons and together these effects contribute to a robust neuroprotective effect in the postischemic brain (summarized in Supplementary Figure S4). Although efforts have been made to develop new therapeutics based on EP, this study represents the first systematic approach to generate and evaluate EP isosteres and present a potential candidate molecule.

Regarding the mechanism underlying its anti-inflammatory effect, our findings indicate DEOPA inhibits both IκB-α degradation in cytosol and p65 to DNA binding in the nuclei of microglial cells (Fig. 6), which stands in contrast to the mechanism of EP, that involves the blockage of p65 to DNA binding but not IκB-α degradation^10^. Accordingly, these two mechanisms probably explain the robust anti-inflammatory effect observed for DEOPA *in vivo* and *in vitro,* which was also considerably greater than EP (Figs 4 and 5). Regarding the molecular mechanism underlying inhibition of p65 to DNA binding by DEOPA, we observed direct inhibition of this binding under cell free conditions (Fig. 6C). However, it considered probable that additional mechanisms might also be involved, such as, covalent modification of p65 at Cys^38^ 10, GSH-depletion-mediated redox state change of p65 9, or the inhibition of ROS-dependent STAT signaling^31^, as have been reported for EP. We speculate that the mechanisms responsible for the robust anti-inflammatory effect of DEOPA are likely to be multifactorial and regardless of the nature of the mechanisms involved, DEOPA might be more potent than EP. Interestingly, DEOPA failed to reduce HMGB1 release in LPS-treated BV2 cells (Supplementary Figure S2), whereas EP has been used to inhibit HMGB1 release and this inhibition contributes to its anti-inflammatory effect^8^,^12^,^32^,^33^. We suggest structure-functional studies are needed to be undertaken to explore the effects of the functional groups of DEOPA on the inhibitions of NF-κB signaling or HMGB1 release.

In the present study, we found that suppression of NF-κB activity by DEOPA not only inhibits microglia activations but suppresses neutrophil infiltration and excitotoxicity in neurons (Figs 4, 7 and 8). Although microglia are the major resident immune cells and play important roles in postischemic inflammation, circulating immune cells that infiltrate brain parenchyma, especially after blood brain barrier (BBB) disruption, also play pivotal roles. Stroke is well-known to be associated with acute and massive influxes of neutrophils, which are the first leukocytes to accumulate around injured brain tissues and aggravate tissue injury^34^. In the present study, we suggested inhibition of the NF-κB activation, and thus the expressions of cell adhesion molecules, by DEOPA underlies effective blocking of neutrophil infiltration into the cortical penumbra of the postischemic brain (Fig. 4). Accumulating evidence indicates an association exists between the proinflammatory activation of neutrophils and infarct size, BBB disruption, hemorrhagic transformation, and poorer neurologic outcomes in the postischemic brain^35^,^36^,^37^. Moreover, activation of NF-κB has known to be involved in NMDA-induced neuronal apoptosis in rat striatum and in primary cortical cultures^30^,^38^. Accordingly, it appears the neuroprotective effect of DEOPA is attributable to its anti-inflammatory effects on microglia and neutrophils and anti-excitotoxic effect on neurons and suppression of NF-κB is critical for both effects.

Regarding the structure-function relationships of EP, some functions appear to be specifically associated with EP and not pyruvate, whereas others are common to both, which suggests that these other functions are due to the pyruvate generated by the de-esterification of EP or to the pyruvoyl moiety of EP. Direct ion chelation, such as, of Ca^2+^ or Zn^2+^, and Nrf2 activation are examples of EP-specific functions^8^,^14^,^15^, whereas NAD replenishment and scavenging hydroxyl radical are examples of the latter^15^,^39^. However, such a classification is incomplete for some functions. For DEOPA, since it cannot be metabolized to pyruvate the observed effects of DEOPA might not be derived from pyruvate itself. In this regard, it is interesting to note that in contrast to EP, DEOPA had no protective effect in primary neuronal cultures exposed to Zn^2+^ (Supplementary Figure S1) and failed to decompose H_2_O_2_ under cell free conditions (Supplementary Figure S3). These findings demonstrate the pharmacologic properties of DEOPA and EP intrinsically differ, and suggest the robust protective effects of DEOPA are due to quite different underlying mechanisms.

In addition to reports issued on commercially available EP-related compounds^20^,^21^, recently, Min *et al*.^40^ reported on the anti-inflammatory effect of EOP (S-ethyl 2-oxopropanethioate) in LPS-treated BV2 cells. However, in the present study, we found that the neuroprotective effect of EOP was unimpressive in the postischemic brain (Fig. 1B). Although more remains to be done to develop improved therapeutics, one important point raised by the above-mentioned studies is that lipophilicity may critically affect therapeutic efficacy^20^,^21^. In terms of the lipophilicities of EP isosteres, the ClogP value of DEOPA is 0.126, which is comparable to that of EP, 0.161, and thus, the comparatively high lipophilicity of DEOPA might also contribute to its neuroprotective effects.

In view of our finding of the substantially greater protective effect of DEOPA than EP in the postischemic brain, we believe DEOPA has unknown functions that contribute to its effects. We suggest further studies be conducted to identify these functions and to elucidate the mechanisms involved.

## Materials and Methods

### General procedure for synthesis of bioisosteres of EP

2-Oxopropanoyl chloride (2), which was prepared from pyruvic acid (1) and α,α-dichloromethyl methyl ether, was added to a solution of ethylamine (1a), diethylamine (1b), or ethanethiol (1c) in dichloromethane (40 mL) at 0 °C. Mixtures were stirred at room temperature for 4 hrs and then quenched with 1 N HCl. Solutions were extracted with ethyl acetate and organic layers were washed with water, brine, dried over Na_2_SO_4_, and evaporated in vacuum. Crude residues were purified by column chromatography to give the title compounds.


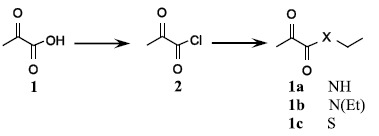


### *N*-Ethyl-2-oxopropanamide (1a)

The title compound was obtained as a yellow oil (620 mg, 19%). Spectral data were consistent with previously reported data^41^.

### *N,N*-Diethyl-2-oxopropanamide (1b)

The title compound was obtained as a yellow oil (650 mg, 16%). Spectral data were consistent with previously reported data data^41^.

### *S*-Ethyl 2-oxopropanethioate (1c)

The title compound was obtained as a yellow oil (1.11 g, 45%). Spectral data were consistent with the previously reported data data^41^.

### hexane-2,3-dione

The compound was purchased from Sigma-Aldrich (Sigma-Aldrich, St. Louis, MO).

### Intravenous administration of EP bioisosteres

Each EP bioisoster was dissolved in 0.01 M PBS. For EP, a solution containing EP (28 mM), Na^+^ (130 mM), K^+^ (4 mM), Ca^2+^ (2.7 mM), and Cl^**−**^ (139 mM) (pH 7.0) was used. DEOPA was administered i.v. at 1, 5, or 10 mg/kg body weight and other EP bioisosteres were administered i.v. at 5 mg/kg body weight EP was administered i.v. at 5 or 10 mg/kg body weight.

### Surgical procedure for MCAO

Male Sprague-Dawley rats (8–9 weeks) were housed under diurnal lighting conditions and provided with food and tap water ad libitum. All animal studies were carried out in strict accordance with the Guide for the Care and Use of Laboratory Animals published by the National Institute of Health (NIH, USA 2013) and complied with ARRIVE guidelines (http://www.nc3rs.org/ARRIVE). The animal protocol used in this study was reviewed and approved beforehand by the INHA University-Institutional Animal Care and Use Committee (INHA-IACUC) with respect to ethicality (Approval Number INHA-140522-297). MCAO was carried out as previously described^42^. In brief, rats (250~300 g) were anesthetized with 5% isoflurane in a 30% oxygen/70% nitrous oxide gas mixture; anesthesia was maintained during procedures using 0.5% isoflurane in the same gas mixture. Animals were randomly allocated to 8 treatment groups, as follows; (1) the MCAO + PBS group; PBS-treated MCAO animals (n = 36), (2) the MCAO + EOPA (N-ethyl-2-oxopropanamide) group; EOPA-treated MCAO animals (n = 10); (3) the MCAO + DEOPA (N,N-diethyl-2-oxopropanamide) group; DEOPA-treated MCAO animals (n = 66); (4) the MCAO + EOP (S-ethyl-2-oxopropanethioate) group; EOP-treated MCAO animals (n = 5); (5) the MCAO + HD (hexane-2,3-dione) group; HD-treated MCAO animals (n = 4); (6) the MCAO + EP group; EP-treated MCAO animals (n = 45); (7) a Sham group; animals underwent surgery but were not subjected to MCAO (n = 22); or (8) the Sham + DEOPA group; DEOPA-treated sham control (n = 5). MCAO was maintained for 1 hr using a nylon suture and this was followed by reperfusion for up to 14 days. In each animal, the left femoral artery was cannulated for blood sampling to analyze pH, PaO_2_, PaCO_2_, and blood glucose concentrations (I-STAT; Sensor Devices, Waukesha, WI). Regional cerebral blood flow (rCBF) was monitored at 1 hr post-MCAO using a laser Doppler flowmeter (Periflux System 5000; Perimed, Jarfalla, Sweden). A thermoregulated heating pad and a heating lamp were used to maintain a rectal temperature of 37.0 ± 0.5 °C during MCAO.

### Infarct volume assessments

Rats were decapitated at 2 days post-MCAO, and whole brains were dissected coronally into 2-mm slices using a metallic brain matrix (RBM-40000, ASI, Springville, UT). Slices were immediately stained by immersing them in 2% 2,3,5-triphenyl tetrazolium chloride (TTC) at 37 °C for 15 min and then fixed in 4% paraformaldehyde. Infarcted tissue areas were quantified using the Scion Image program (Frederick, MD). To account for edema and shrinkage, areas of ischemic lesions were calculated using (contralateral hemisphere volume – (ipsilateral hemisphere volume – measured injury volume)). Infarct volumes were quantified (in mm^3^) by multiplying summed section infarct areas by section thickness.

### Modified neurological severity scores (mNSS)

Neurological deficits were evaluated using mNSSs at 2, 5, 7, 10, or 14 days post-MCAO. The mNSS system consists of motor, sensory, balance and reflex tests and overall results are graded using a scale of 0 to 18 (normal: 0, maximal deficit: 18)^43^.

### Rota-rod test

Twenty-four hours before surgery, rats were conditioned on a rota-rod unit at a constant speed (3 rpm) until they were able to remain on the rotating spindle for 180 s. At 2, 5, 7, 10, or 14 days post-MCAO, rats were subjected to rota-rod testing at spindle speeds of 5 and 15 rpm and residence times on the spindle were recorded with a 1 hr rest period after each test.

### Immunohistochemistry

Immunological staining of brain sections was performed using a floating method, as previously described^44^. Primary antibodies were diluted as follows; 1:500 for anti-ionized calcium binding adaptor molecule-1 (Iba-1) (Wako Pure Chemicals, Osaka, Japan), 1:250 for anti-Mac-2 (Abcam, Cambridge, UK), and 1:500 for anti-myeloperoxidase-1 (MPO-1) (Abcam, Cambridge, UK). Experiments were repeated at least three times and representative images are presented.

### Cell cultures

BV2 cell (a microglial cell line), human umbilical vein endothelial cells (HUVECs), and HL-60 cells (a human myelocytic leukemia cell line) were obtained from the American Type Culture Collection (ATCC, Rockville, MD). BV2 cells were cultured as previously described^14^. HUVECs were cultivated in Endothelial Cell Medium (ScienCell, Corte Del Cedro Carlsbad, CA). HL-60 cells were cultivated in DMEM supplemented with 20% FBS and 100 U/ml penicillin/streptomycin. HL-60 differentiation was induced by treating 1 μM all-trans retinoic acid (ATRA) (Sigma-Aldrich, St. Louis, MO) for 3 days. All these cell types were incubated at 37 °C in a 95% air/5% CO_2_ humidified atmosphere.

### NO measurements

Nitrite production in BV2 cells (1.5 × 10^5^) were measured as described^14^.

### RNA preparation and quantitative PCR

RNA was prepared using TRIzol reagent (Gibco BRL, Gaithersburg, MD), and 1 μg aliquots were used for cDNA synthesis, which was conducted using a RT-PCR kit (Roche, Mannheim, Germany). The sequences of the rat tumor necrosis factor-α (TNF-α), interleukin-1β (IL-1β), cyclooxygenase-2 (Cox-2), inducible NO synthase (iNOS), and GAPDH primers used were described previously^14^.

### Nuclear and Cytoplasm Extract Preparation

Nuclear extracts were prepared using Nuclear Extraction Kits (IMGENEX, San Diego, CA). BV2 cells (5 × 10^6^) and brain tissues were used for nuclear/cytoplasm extract preparation and crude nuclear proteins in supernatants were stored at −70 °C after collection.

### Immunoblot analysis

Proteins (20 μg) were separated in 12% sodium dodecyl sulfate-polyacrylamide gels. After blocking with 5% non-fat milk for 1 hr, membranes so obtained were incubated with primary antibodies (all diluted 1:1000) for anti-IκB-α (Santa Cruz Biotechnology, Santa Cruz, CA), anti-α-tubulin (Cell Signaling, Danvers, MA), anti-p65 (Santa Cruz Biotechnology), anti-Lamin B (Santa Cruz Biotechnology), anti-P-selectin (Santa Cruz Biotechnology), anti-ICAM-1 (Santa Cruz Biotechnology), anti-PSGL-1 (Santa Cruz Biotechnology), and anti-CD11b (BD Transduction Laboratories, San Jose, CA) overnight at 4 °C. The next day, blots were detected using a chemiluminescence kit (Roche, Basel, Switzerland) using HRP-conjugated secondary antibodies (1:2000; Santa Cruz Biotechnology).

### NF-κB binding activity assay

Nuclear extracts were prepared using Nuclear Extraction Kits (IMGENEX, San Diego, CA). p65 to DNA binding activity was measured using a TransAM NF-κB p65 assay kit (Active Motif, Carlsbad, CA) and recombinant p65 by following manufacturer’s recommendation.

### Transient transfection and the luciferase assay

BV2 cells (1.5 × 10^5^) were seeded in 24-well plates containing DMEM and 1 day later transfected with the a NF-κB reporter plasmid containing five copies of the NF-κB response element that drives transcription of the luciferase reporter gene (Promega, Madison, WI) using Lipofectamine 2000 transfection reagent (Invitrogen, Carlsbad, CA). Transfection procedures and luciferase assay were followed as previously described^14^.

### Cell adhesion and transendothelial migration assays

HUVECs (5 × 10^4^) were seeded in 24-well plates and grown for 24 hrs to form monolayers. Before adhesion assay, HUVECs and HL-60 cells were labeled for 30 min with CellTracker™ Green CMFDA or CellTracker Red CMTPX Dye (Invitrogen, Carlsbad, CA), respectively. Cells were treated with DEOPA (5, 10 mM) or EP (10, 20 mM) for 1 hr and treated with TNF-α (10 U/ml) for 12 hrs for activation. For adhesion assay, dHL-60 cells (10^5^) were added to HUVEC monolayers for 30 min at 37 °C, washed twice with ice-chilled 0.01 M PBS, and fixed with 4% paraformaldehyde. For transendothelial assay, HUVECs (1 × 10^4^) were grown to confluence (48 hrs) on the 5.0 μm pore-sized, gelatinized polycarbonate membrane of the upper chamber of a Boyden chamber (SPL, Gyeonggi-do, Korea) and the labeled dHL-60 cells in migration buffer (serum free DMEM) were added on the monolayer of HUVECs and incubated for 2 hrs. The number of attached or migrated cells to the lower face of the filter was counted from 12 photographs taken during three independent experiments.

### Primary cortical neuron culture and NMDA treatment

Mixed cortical cultures were prepared as described previously^42^ and 30 μM NMDA (Sigma, St. Louis, MO) were treated for 10 min with serum-free MEM and media were then removed and replaced with fresh MEM medium, and cells were cultured for a further 24 hrs.

### Measurement of NAD Levels

Cells were extracted in 0.25 ml of 0.5 N HClO_4_, neutralized with 3 M KOH/125 mM GlyeGly buffer (pH 7.4), and centrifuged for 5 min at 10,000 g. Brain tissues were isolated from cortex of each animal groups and treated with 0.5 M perchloric acid (Sigma-Aldrich) for 15 min at 4 °C and homogenized. NAD concentrations were determined using a cyclic enzymatic assay^45^.

### Statistical analysis

Two-sample comparisons were performed using the Student’s t test and multiple comparisons by one-way or two-way analysis of variance (ANOVA) followed by post hoc testing. PRISM software 5.0 (Graph Pad Software) was used for the analysis. Results are presented as means ± SEMs and statistical difference was accepted for p values < 0.05.

## Additional Information

**How to cite this article:** Lee, H.-K. *et al*. Anti-inflammatory and anti-excitoxic effects of diethyl oxopropanamide, an ethyl pyruvate bioisoster, exert robust neuroprotective effects in the postischemic brain. *Sci. Rep.* **7**, 42891; doi: 10.1038/srep42891 (2017).

**Publisher's note:** Springer Nature remains neutral with regard to jurisdictional claims in published maps and institutional affiliations.

## Supplementary Material

Supplementary Information
