# Supplementary material for: Anti-inflammatory and anti-excitoxic effects of diethyl oxopropanamide, an ethyl pyruvate bioisoster, exert robust neuroprotective effects in the postischemic brain
Source: Sci Rep. 2017 Feb 21;7:42891. doi: 10.1038/srep42891 (PMC5318887; doi:10.1038/srep42891)
Supplement: Supplementary Information [file srep42891-s1.doc]

Supplementay information

Anti-inflammatory and anti-excitoxic effects of diethyl oxopropanamide, an ethyl pyruvate bioisoster, exert robust neuroprotective effects in the postischemic brain

# Hye-Kyung Lee^1,2#^, Il-Doo Kim^1,2#^, Seung-Woo Kim^1,3^, Hahnbie Lee ^1,2^, Ju-Young Park^4^, Sung-Hwa Yoon^4*^, Ja-Kyeong Lee^1,2*^

^1^Department of Anatomy, ^2^Medical Research Center, ^3^Department of Biomedical Sciences, Inha University School of Medicine, Inchon, Korea; ^4^Department of Molecular Science and Technology, Ajou University, Suwon, Republic of Korea

#

# ^*^ They are equally contributed

# ^*^ Corresponding author: jklee@inha.ac.kr

# Table S1

**Table S1. Physiological parameters after treatment of EP bioisostere, DEOPA**

Values are means ± SD (n=5). One-way Analysis of variance revealed no significant intergroup difference for any variance.

#
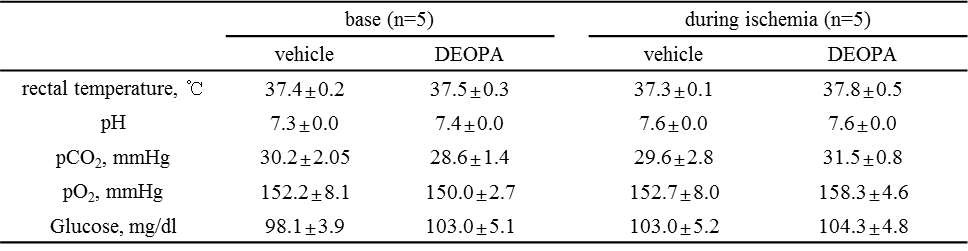


**Fig. S1**

**
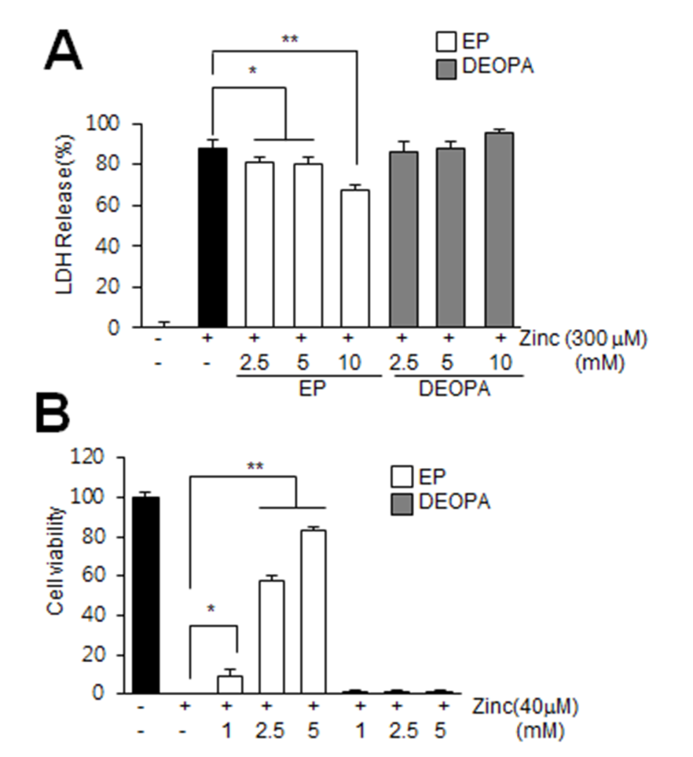
**

**Figure S1. EP but not DEOPA exhibited Zn^2+^-induced toxicity in primary cortical culture**

LDH levels in primary cortical cultures (4ⅹ10^5^ cells/well in 24-well culture dishes) were measured 24 hrs after treating cells with Zn^2+^ (300 μM, 30 min) (A) or with Zn^2+^ (40 μM, 24 hrs) in the presence of DEOPA or EP (2.5, 5, or 10 mM or 1, 25., or 5 mM of each). Results are presented as means±SEMs. * *p* < 0.05 **, *p* < 0.01 vs. Zn^2+^-treated control.

**Fig. S2**

**
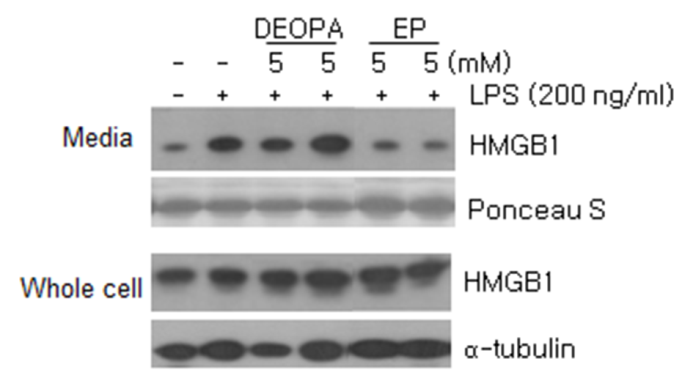
**

**Figure S2. EP but not DEOPA blocked HMGB1 secretion in activated microglia**

Primary microglial cultures (3×10^5^ cells/well) were incubated with 200 ng/ml of LPS in the presence or absence of EP or DEOPA (5 mM) for 24 hrs and levels of HMGB1 in cell homogenates and culture media were then determined by immunoblotting with anti-HMGB1 antibody.

**Fig. S3**

**
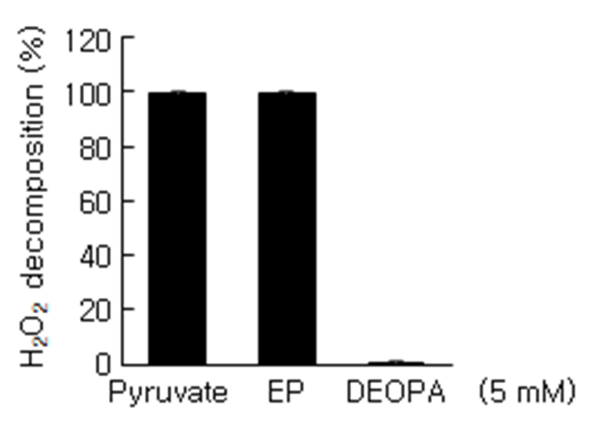
**

**Figure S3.** **H_2_O_2_ decomposition by EP or DEOPA under cell free conditions**

The capacity of hydrogen peroxide decomposition of EP (5 mM), pyruvate (5 mM), or DEOPA (5 mM) was measured after 5 min of incubation in reaction solution. The absorbance of the mixture was measured at 350 nm.

**Table S2. Spectral data of EP isosteres**

| **EP isosteres** | Spectral data |
| --- | --- |
| ***N*-Ethyl-2-oxopropanamide**  **(EOPA)** | IR (KBr) cm^-1^: 1720, 1686; ^1^H nuclear magnetic resonance (NMR) (CDCl_3_) δ: 1.15-1.25 (t, 3H, *J* = ­7.2 Hz), 2.47 (s, 3H), 3.30-3.40 (q, 2H, *J* = 6.4 Hz); ^13^C-NMR (CDCl_3_) δ: 14.55, 24.52, 34.33, 159.75, 196.98; MS *m/z*: 116.00 ([M+H]^+^) |
| ***N*,*N*-Diethyl-2-oxopropanamide**  **(DEOPA)** | IR (KBr) cm^-1^: 1716, 1634; ^1^H-NMR (CDCl_3_) δ: 1.15-1.25 (m, 6H), 2.42 (s, 3H), 3.25-3.30 (q, 2H, *J* = 6.8 Hz), 3.35-3.45 (q, 2H, *J* = 7.2 Hz); ^13^C-NMR (CDCl_3_) δ: 12.54, 14.41, 27.52, 39.32, 41.95, 166.07, 198.37; MS *m/z*: 143.90 ([M+H]^+^) |
| ***S*-Ethyl 2-oxopropanethioate**  **(EOP)** | IR (KBr) cm^-1^: 1724, 1669; ^1^H-NMR (CDCl_3_) δ: 1.25-1.34 (t, 3H, *J* = 7.6 Hz), 2.42 (s, 3H), 2.90-3.00 (q, 2H, *J* = 7.6 Hz); ^13^C-NMR (CDCl_3_) δ: 14.02, 22.90, 23.69, 190.64, 192.64; MS *m/z*: 132.80 ([M+H]^+^) |

**Fig. S4**


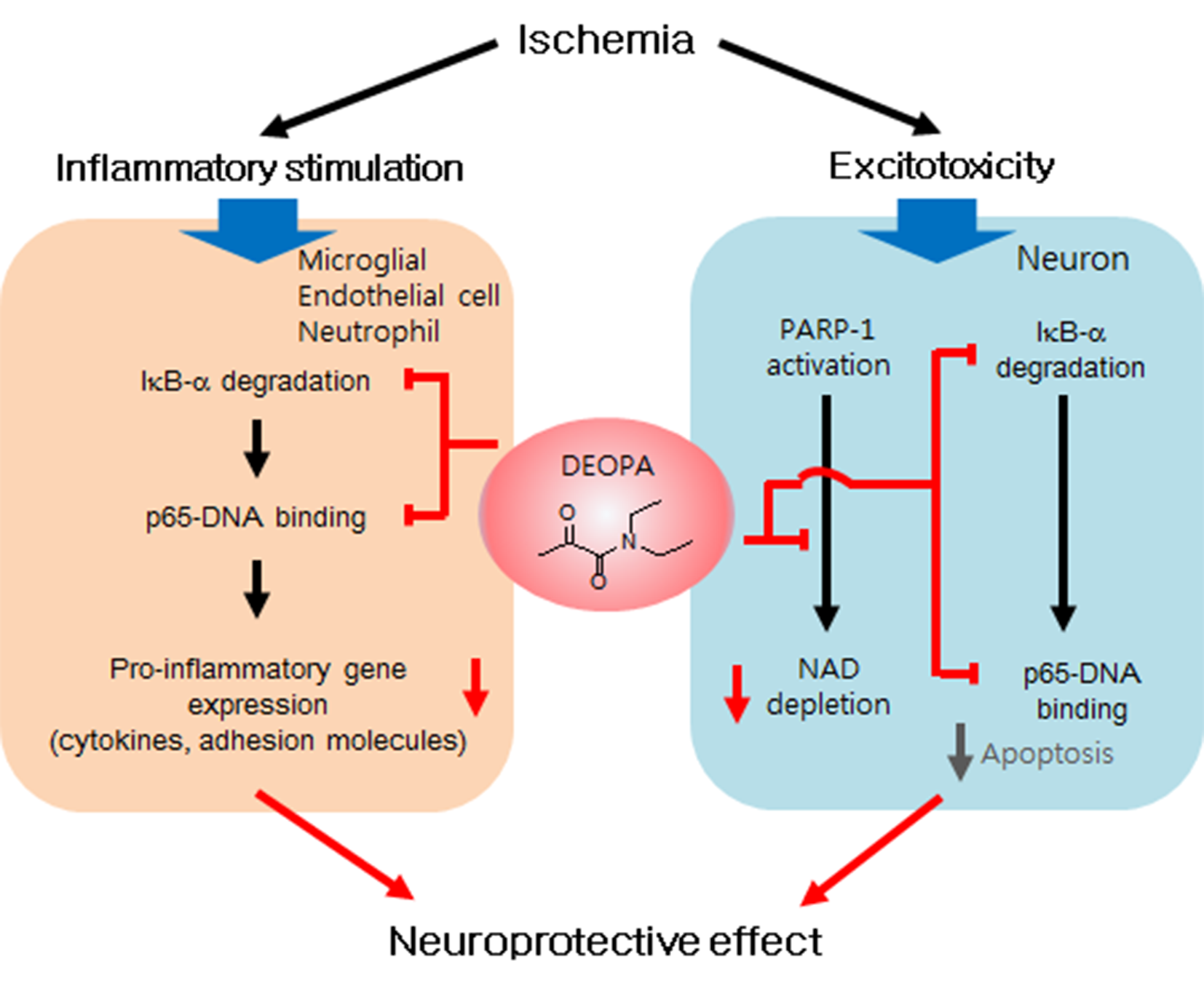


**Figure S4. Mechanism of multi-modal neuroprotectant, DEOPA**

Schematic diagram shows the neuroprotective effects of DEOPA via anti-inflammation and anti-excitotoxicity.
